# Evaluation of the 'healthy start to pregnancy' early antenatal health promotion workshop: a randomized controlled trial

**DOI:** 10.1186/1471-2393-12-131

**Published:** 2012-11-19

**Authors:** Shelley A Wilkinson, H David McIntyre

**Affiliations:** 1 Mater Medical Research Institute, Mothers and Babies Theme, Raymond Terrace, South Brisbane, Queensland, 4101, Australia; 2 Department of Nutrition and Dietetics, Level 3 Mater Children's Hospital, Raymond Terrace, South Brisbane, Queensland, 4101, Australia; 3 University of Queensland, Mater Clinical School, South Brisbane, Queensland, 4101, Australia

**Keywords:** Antenatal, Behavior change, Fruit, Health service delivery, Nutrition, Physical activity, Pregnancy, Vegetables

## Abstract

**Background:**

Pregnancy is an ideal time to encourage healthy lifestyles as most women access health services and are more receptive to health messages; however few effective interventions exist. The aim of this research was to deliver a low-intensity, dietitian-led behavior change workshop at a Maternity Hospital to influence behaviors with demonstrated health outcomes.

**Methods:**

Workshop effectiveness was evaluated using an RCT; ‘usual care’ women (n = 182) received a nutrition resource at their first antenatal visit and 'intervention' women also attended a one-hour ‘Healthy Start to Pregnancy’ workshop (n = 178). Dietary intake, physical activity levels, gestational weight gain knowledge, smoking cessation, and intention to breastfeed were assessed at service-entry and 12 weeks later. Intention-to-treat (ITT) and per-protocol (PP) analyses examined change over time between groups.

**Results:**

Approximately half (48.3%) the intervention women attended the workshop and overall response rate at time 2 was 67.2%. Significantly more women in the intervention met pregnancy fruit guidelines at time 2 (+4.3%, *p = 0.011*) and had a clinically-relevant increase in physical activity (+27 minutes/week) compared with women who only received the resource (ITT). Women who *attended* the workshop increased their consumption of serves of fruit (+0.4 serves/day, *p = 0.004*), vegetables (+0.4 serves/day, *p = 0.006*), met fruit guidelines (+11.9%, *p < 0.001*), had a higher diet quality score (*p = 0.027*) and clinically-relevant increases in physical activity (+21.3 minutes/week) compared with those who only received the resource (PP).

**Conclusions:**

The Healthy Start to Pregnancy workshop attendance facilitates improvements in important health behaviors. Service changes and accessibility issues are required to assist women's workshop attendance to allow more women to benefit from the workshop’s effects.

**Trial registration:**

Australian New Zealand Clinical Trials Registry ACTRN12611000867998

## Background

Pregnancy health behaviors are associated with pregnancy-related and long-term health outcomes for both the mother and infant. Cigarette smoking [1], poor nutrition [2-4], insufficient levels of physical activity (PA) [5],and awareness of gestational weight gain GWG) goals [6,7] have been associated with a number of poor outcomes, including an increased risk of caesarean sections [8], low birth weight [1], pre-term birth [1,8], inappropriate GWG [9], and chronic disease in adult life [2,5,8-10]. Adherence to health behavior recommendations during pregnancy has been shown to improve pregnancy outcomes [3,4], including decreasing the risk of gestational diabetes mellitus (GDM) [11], pre-eclampsia [3], physical pregnancy symptoms (e.g. back pain, nausea etc.) [12], and improved mental health [13]. However, low levels of adherence to pregnancy health behavior recommendations have been demonstrated in a number of Australian pregnant populations [14-16]. Furthermore, Queensland (Australia) breastfeeding rates are below National targets [17], suggesting a need for intervention to improve health behaviors and subsequent outcomes.

Pregnancy is an ideal time to implement health behavior changes. The majority of women are in contact with the health service for antenatal care [18] and are more receptive to health messages [19,20]. Current guidelines recommend that all pregnant women should receive advice about the important factors which may influence pregnancy outcomes [21]. Women may receive lifestyle information via antenatal classes. However, these classes are often conducted late in pregnancy and mainly focus on birth and labour, rather than facilitating healthier lifestyles.

Provision of population-based guidelines alone is not effective for behavior change [22]. Limited literature exists about effective methods to deliver pregnancy-related healthy lifestyle information [14], although, in general, individual and group health education approaches are both effective [23]. Groups are more cost effective, but should have a theoretical base (e.g. [24]) to facilitate behavior change [23]. An evidence-based self-management framework, such as 5As (assess, advise, agree, assist, arrange), can assist health professionals in supporting and guiding patients' self-directed behavior change [25] and is an ideal structure to deliver an low-intensity antenatal health promotion with a focus on improving health behaviors.

In summary, there is evidence that women require information about important health behaviors during pregnancy and assistance in meeting population health guideline targets. Our research aim was to evaluate the effectiveness of the delivery of low-intensity early antenatal health promotion program ('The Healthy Start to Pregnancy; HSP) designed in line with the 5As and behavior change principles on improving maternal health behaviors at our tertiary Maternity Hospital (MH). Our primary hypothesis was that HSP attendance would improve dietary behaviors, as assessed by a between group difference change in meeting fruit and vegetable pregnancy guidelines by 5%, daily fruit and vegetables of half a serve each, an improved diet quality index, and improved GWG guideline awareness, compared with usual care. Our secondary hypotheses were that HSP attendance would result in an increase, by at least 5%, of women undertaking adequate levels of PA; an increase of at least 30 minutes of PA per week; a decrease of at least 5% in the percentage of women who smoked during pregnancy; and an increased proportion of maternal intention to breastfeed.

## Methods

### Design and participants

The HSP workshop was evaluated using a randomized controlled trial (RCT) design, comparing ‘usual care’ (UC) and ‘intervention’ (HSP) arms in a Tertiary MH service in South East Queensland (Australia) with approximately 5,000 births a year. Women were eligible if they were attending their booking visit at our MH research site and were 18 years or older (or under 18years, with the consent of a parent or guardian). Women were excluded if they were unable to read and speak English at a level that allowed completion of pen-and-paper surveys.

Data collection occurred at two time points; Time one = booking visit (~14 weeks of pregnancy) and Time two = +12weeks post-service entry (~26 weeks of pregnancy) following an adapted Dillman postal survey method, with a reminder letter, survey and second survey posted at two-week intervals [26]. The first data collection point was at recruitment in clinic and the second was by postal survey. Recruitment ran from 31 August 2010 to 7 March 2011.

The sample size required to detect hypothesized differences between groups in relevant health behaviors was based on previous research for intervention effects on each of the behaviors being targeted [27]. To detect hypothesized 5% differences between groups in the prevalence of each of the targeted health behaviors required 129 participants per group providing 80% power and two-sided α set at 0.05 [28,29]. To detect a between-group difference of half a daily fruit serve and half a daily vegetable serves required 64 participants per group. To detect the hypothesized minimum between-group difference of 30, 60 or 90 minutes per week in PA required 319, 143 or 81 participants per group, respectively [11,15,27,30-32]. Allowing for a non-consent and attrition rate of 20%, approximately 360 women needed to be recruited (180 in each group) to be able to detect most of the hypothesized outcomes. Approximately 340 women enter the MH service every month, thus we estimated capacity to recruit 70 to 100 women per month over a four to five month study period, assuming a 40-60% consent rate (based on previous research [27]).

### Procedures

Women were invited to participate by research assistants trained in ethical conduct for research. Those who provided consent were randomised to the usual care (UC) or intervention (HSP) group. The computerized randomisation process was managed by the research hospital’s clinical research support unit; allocation was concealed using sealed opaque envelopes. Eligible women were identified and approached by the research officer at their booking visit antenatal clinic (ANC) appointment. Those who consented to participation completed baseline data collection at time of consent and a group booking was made for those in the HSP group. Reasons for refusal were recorded. The study was explained as a trial evaluating different ways to support healthy lifestyles during pregnancy. Women in the HSP group were also required to attend a 60 minute group session at a suitable time (morning, afternoon and evening times available) [14].

### Outcome measures

Rather than measure actual GWG (due to the complexity of it being an intermediate step as a reflection of lifestyle behaviours, as well as a potential predictor of pregnancy outcome) our attempt has been to address the ‘upstream’ behaviours of good nutrition, physical activity and knowledge of guidelines to influence GWG. Outcome measures were average daily serves of fruit and vegetables (primary), diet quality index (primary), weekly minutes of physical activity (secondary), number of cigarettes smoked (secondary), the percentage of women meeting health behavior guidelines (fruit, vegetables (primary), physical activity, and smoking (secondary)), and awareness of GWG guidelines (i.e. the GWG range they should aim for, based on their pre-pregnancy BMI) (primary). Intention to breastfeed (secondary) and pre-pregnancy BMI was also collected. Measures were self-reported using valid and reliable self-report measures of the health behaviors of interest (smoking status (Smoke-Free Families Common Evaluation Measures for Pregnancy and Smoking Cessation) [33,34]; fruit and vegetable intake (National Nutrition Survey Fruit and Vegetable Questions) [35,36], and diet quality index (Fat and Fibre Behaviour Index) [37]; physical activity (Active Australia Questionnaire) [31]; breastfeeding intention (Infant Feeding Intentions Scale) [38]). Current weight and height was self-reported, due to the high correlation between measured and self-report anthropometry [39]. Body Mass Index (BMI) was calculated from pre-pregnancy weight (kg) and height (m). Australian Bureau of Statistics Population Statistics Group standards for the collection of demographic characteristics were used ( http://www.abs.gov.au). Pregnancy history information was collected; parity (P) was determined by asking ‘*How many times have you been pregnant (that has resulted in a live birth)*’, and gestation (G) by asking ‘*Including this pregnancy, how many times have you been pregnant?’*. We also measured women’s group attendance.

### Ethics

This research was approved by the Mater Health Services Human Research Ethics Committee (1465M). This study conformed to procedures in accordance with ethical standards on human experimentation.

### Usual care and intervention delivery

#### Usual care - healthy eating during pregnancy booklet

Women in the UC arm received usual (nutrition) care through the MH. Prior to the research trial, a new booklet was introduced to the ANC that was distributed to all women at their booking (first) visit with a midwife (replacing out-dated and incorrect A5-sized flyers). This new resource was compiled according to best-practice for health education print material [40,41] and contained evidence-based (EB) literature regarding behaviors that influence maternal and infant health outcomes (fruit and vegetable intake; healthy weight gain; physical activity) [3-5,12,42,43] that facilitated health behavior change [24]. The design and content of the booklet was also informed by sources that included (i) *The Pregnancy Pocketbook*[23]; (ii) findings from Wilkinson and Miller [44]; and (iii) women’s feedback [10]. The resource was a 12-page booklet, with EB information, screening tools (GWG, fruit and vegetable intake), goal setting and self-monitoring activities (GWG, recommended fruit and vegetable intake), and referral information (Dietitian and Physiotherapist). The booklet had a Flesch-Kincaid readability score of 7.9, meeting the recommendation of printed health information being less than 8 (equivalent to grade 8 or the 8^th^ year of schooling).

#### Intervention – HSP workshop and healthy eating during pregnancy booklet

The 60 minute HSP session (capacity 15 women (+/− partners)) was designed, by the author (SW), according to the 5As and was delivered by maternity dietitians from the MH Nutrition & Dietetic department experienced in adult learning principles. Extensive multidisciplinary (midwifery, physiotherapy, occupational therapy, social work) input was obtained in development of group content. It was delivered according to a session plan and PowerPoint slides to facilitate information sharing, discussion and activities to extend the booklet information. The session plan included: 1. delivery of valid and reliable screening tools to identify women at-risk of not meeting health behavior guidelines for pregnancy (dietary advice (fruit and vegetable intake), healthy weight gain, smoking cessation, physical activity) (‘Assess’), 2. delivery of EB nutrition and physical activity information and behavior change strategies, including an explanation of and assistance with goal setting and self-monitoring, tailored to behaviors identified in the ‘assess’ activity with personalised activity sheets to allow recording of individual goals and mapping of behaviours, for pregnancy (designed to improve self-efficacy [24]) (‘Advise’, ‘Agree’, “Assist’) and, 3. providing women with links to more specialised services in supporting behavior change, where required (‘Arrange’).

### Statistics

Quantitative data were analysed with SPSS for Windows version 15 (SPSS, Chicago, Illinois) and StataSE version 10.1 (StataCorp Pty Ltd, College Station, Texas, United States of America). Means and standard deviations (normal distributions), medians and interquartile ranges (IQR) (skewed distributions) or frequencies were calculated. 2009 IOM GWG guidelines [42] were used in analyses. An extra coding category of '*rounded (up or down)*' (GWG goal rounded up to the nearest whole number) was created when assessing GWG knowledge. This extra analysis of GWG knowledge was included following clinical observations that some women would discuss their ‘rounded’ GWG goal with clinicians. For example, a healthy weight woman would need to gain between 11.5 to 16kg for pregnancy. We wanted to assess the proportion of women who rounded to 12 to 16 or 11 to 16kg, being unaware of the precision of the guidelines or our study protocol. Fruit and vegetable intake was assessed against Australian Guide to Healthy Eating (AGHE) recommendations for pregnancy (four and five serves daily, respectively) [43]. Minutes of physical activity per week were assessed against The American College of Obstetrics and Gynecology’s physical activity guidelines [45] which recommend at least half an hour of moderate exercise on most, if not all days during pregnancy (equating to 150 minutes per week), and mirror physical activity guidelines for the general Australian population [31](in lieu of Australian pregnancy guidelines).

Intention-to-treat (ITT) and per-protocol (PP) analyses were used to examine change in behaviors over time between groups. Change variables were calculated for all behaviors. Difference from time 1 to time 2 was calculated for continuous variables. Categorical variables were constructed reflecting proportion of women who met behavioral guidelines at each time point. Differences over time in whether women continued to meet or not meet guidelines were calculated. Change variables for continuous measures were checked and met normality assumptions. Differences were examined with independent group t-tests and independent group χ^2^ tests, including Fishers Exact tests for outcomes with cells <5, and Mann U Whitney tests (for baseline non-parametric variables).

## Results

### Participants

**Figure 1:**
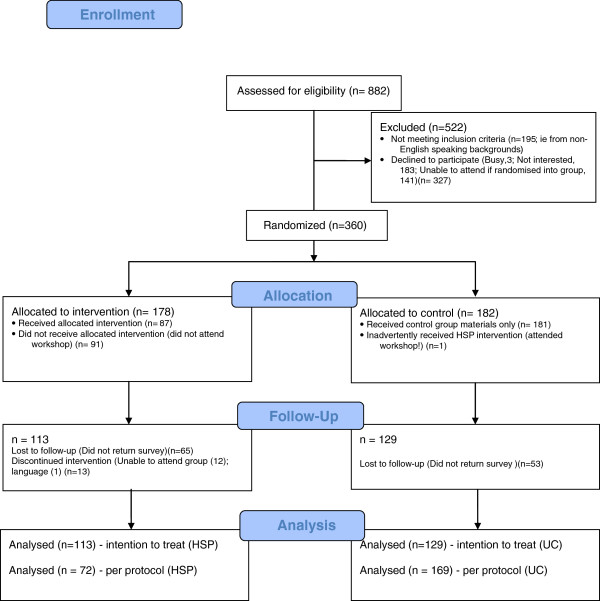
Consort diagram outlining study recruitment and retention.

Three hundred and sixty (178 HSP, 182 UC; 52.4%) of the 687 eligible women approached were recruited (Figure 1) during the recruitment period. Of the 178 HSP women, 87 (46.5%) attended the intervention; one UC woman also attended the intervention. At time 2, survey return was 63.5% from the HSP arm and 70.9% from the UC arm.

At baseline, no statistically significant differences were observed between the HSP and UC samples on any anthropometric or socio-demographic characteristics (Table 1). Further, no statistically significant differences were observed in any health behaviors or meeting health behavior guidelines (Tables 2 and 3). At recruitment, mean age was 29.3 ± 4.9 years, mean BMI 25.0 ± 5.4 kg/m^2^ and mean gestation at 14.3 ± 2.9 weeks.

**Table 1:** Demographic characteristics of the intervention and control sample

**Characteristic**	**Mean (SD, range)or percentage**	**Mean (SD, range)or percentage**	**Maternity hospital Population**
	**Intervention sample(n = 178)**	**Usual care sample(n** = **182)**	
**Age(years)**	29.5 (5.1;18–44)	29.0 (4.7; 19–41)	29.4 (5.8)
< 20 years	2.8%	2.2%	5.0%
20-24 years	12.9%	15.9%	48.6%
25-29years	34.8%	35.2%	
30-34 years	32.0%	33.5%	41.5%
35-39 years	15.2%	12.6%	
≥ 40	2.2%	0.5%	5.0%
**Pre**-**pregnancy BMI** (**kg**/**m**^**2**^)	25.4 (5.2)	24.6 (5.5)	24.7 *(*6.6)
Underweight (<18.5)	4.5%	6.6%	7.5%
Healthy weight (18.5-24.9)	48.3%	51.6%	55.2%
Overweight (25–29.9)	26.4%	25.3%	19.9%
Obese (≥ 30)	17.4%	14.3%	15.9%
Missing	3.4%	2.2%	1.5%
**Education**:			-
Did not finish high school	27.5%	30.8%	
Trade/apprenticeship/diploma	29.2%	30.2%	-
Degree/higher degree	43.3%	38.5%	-
**Household income** (**gross**, **per year**)			-
<$20,000	1.1%	1.6%	-
$20,001-35000	6.2%	5.5%	-
$35,001-50,000	14.6%	11.0%	-
$50,001-70,000	18.5%	20.9%	-
$70,001-100,000	29.8%	24.2%	-
>$100,000	19.7%	25.3%	-
Refused to answer	5.1%	6.6%	-
Not sure	4.5%	3.3%	-
Missing	0%	1.1%	-
**Marital status**			-
Married/de facto	90.4%	93.4%	-
Separated/Divorced	2.2%	0.6%	-
Never Married	7.3%	5.5%	-
Missing	0%	0.6%	-
**Employment**			-
Full time work	49.4%	52.7%	-
Part time/ Casual work	19.1%	23.1%	-
Studying	6.2%	5.5%	-
Full time home duties	15.7%	11.5%	-
Unemployed	9.6%	6.6%	-
Missing	0%	0.5%	-
**ATSI status**			-
Aboriginal, but not Torres Strait Islander	0.6%	0.5%	-
Neither	99.4%	98.9%	-
Missing	0%	0.5%	-
**Gravida**	2.1 (1.5)	2.0 (1.4)	-
**Parity**	0.6 (0.9)	0.6 (0.8)	-
**Stage of gestation** – **weeks**	14.5 (3.0)	14.2 (3.0)	-

**Table 2:** **Proportion of women meeting health outcomes** (**including changes over time**) **in each study arm** (**ITT and PP analyses**)

**Health behaviors**	**Analysis**	**Baseline difference(HSP - UC)**	**HSP 1(n)**	**HSP2(n)**	**HSP mean difference over time**	**Usual care 1(n)**	**Usual care 2(n)**	**UC mean difference over time**	**Between group difference, time 2: HSP - UC**
**Percentage of women meeting pregnancy fruit guidelines**	ITT*	p = 0.66	8.5 (15)	7.3 (13)	−1.2%	9.9 (18)	4.4 (8)	- 5.5%	+**4**.**3**%, **p** = **0**.**009**
	PP*	-	6.8 (6)	12.5 (11)	+5.7%	9.9 (27)	3.7 (10)	−6.2%	+**11**.**9**%, **p** = **0**.**001**
**Percentage of women meeting pregnancy vegetable guidelines**	ITT	p = 0.60	6.2 (11)	6.2 (11)	0%	4.9 (9)	4.9 (9)	0%	0%, p = 0.75
	PP	-	9.1 (8)	11.4 (10)	+2.3%	4.4 (12)	3.7 (10)	−0.7%	+3.0%, p = 0.13
**Percentage of women with correct GWG knowledge**	ITT	*p = 0.57*	0 (0)	1.1 (2)	+1.1%	0 (0)	1.1 (2)	+1.1%	0%, *p = 0.63*
	PP	-	0 (0)	2.3 (2)	+2.3%	0 (0)	0.7 (2)	+0.7%	+1.6%, *p = 0.36*
**Percentage of women with correct GWG knowledge(‘rounded’)**	ITT	*p = 0.85*	1.1 (2)	8.3 (9)	+8.3%	1.1 (2)	4.8 (6)	+4.8%	+3.5%, *p = 0.25*
	PP*	-	1.1(1)	10.2 (9)	+9.1%	1.1 (3)	2.2 (6)	+1.1%	+**8**.**0**%, ***p****=****0****.****009***
**Percentage of women meeting pregnancy physical activity guidelines**	ITT	*p = .33*	44.9 (80)	37.1 (66)	−7.8%	40.1 (73)	30.8 (56)	−9.3%	−1.5%, *p= 0.66*
	PP	-	51.1 (45)	55.7 (49)	+4.6%	39.7 (108)	26.8 (73)	−12.9%	+17.5%, *p = 0.83*
**Percentage of women smoking before(pre)and during pregnancy**	ITT	*p = 0.97 (pre) p = 0.88*	18.0 (32)(pre) 16.3 (29)	5.1 (9)	−1.7% (pre) - 11.2%	18.1 (33) (pre) 17.0 (31)	7.7 (14)	−1.1% (pre) −9.3%	−2.3%, *p=.41*
	PP	-	11.4 (10) 8.0 (7)	3.4 (3)	+4.6%	20.2 (55) 19.5 (53)	7.4 (20)	+12.1%	- 7.5%, *p=0.37*

* significant difference between groups over time; Differences were examined with independent group χ^2^ tests, including Fishers Exact tests for outcomes with cells <5 (GWG knowledge).

**Table 3:** **Behavioural and dietary measures of women** (**including changes over time**) **in each study arm** (**ITT and PP analyses**)

**Health behaviors**	**Analysis**	**Baseline difference (HSP - UC)**	**HSP 1 (n)**	**HSP 2 (n)**	**HSP mean difference over time**	**Usual care 1 (n)**	**Usual care 2 (n)**	**UC mean difference over time**	**Between group difference, time 2: HSP - UC (95%CI)**
**Mean serves of fruit per day (± SD)**	ITT	p = 0.60	1.9 ± 1.2 (176)	2.2 ± 1.1 (112)	0.3 ± 1.0 (112)	1.8 ± 1.2 (182)	1.9 ± 1.1 (126)	0.2 ± 0.9 (128)	+0.2 (−0.1-0.4), *p= 0.14*
	PP*	-	2.0 ± 1.0 (87)	2.4 ± 1.0 (72)	0.4 ± 1.0 (72)	1.8 ± 1.2 (271)	1.9 ± 1.1 (168)	0.1 ± 0.9 (168)	+**0**.**4** (**0**.**1**-**0**.**6**), ***p****=****0****.****004***
**Serves of vegetables per day (± SD)**	ITT	p = 0.24	2.5 ± 1.3 (177)	2.9 ± 1.3 (112)	0.4 ± 1.1 (112)	2.3 ± 1.3 (182)	2.5 ± 1.3 (129)	0.2 ± 1.1 (129)	+0.2 (−0.1 - 0.5), *p = 0.11*
	PP*	-	2.7 ± 1.4 (87)	3.3 ± 1.3 (72)	0.6 ± 1.1 (72)	2.3 ± 1.2 (272)	2.4 ±1.2 (169)	0.2 ± 1.0 (169)	+**0**.**4** (**0**.**1**-**0**.**7**), ***p****=****0****.****006***
**Diet quality – Fat score (± SD)**	ITT	p = 0.61	3.3 ± 0.5 (174)	3.4 ± 0.5 (109)	0.1 ± 0.4 (108)	3.3 ± 0.5 (180)	3.4 ± 0.5 (126)	0.1 ± 0.3 (126)	−0.02 (−0.07-0.1), *p =* 0.68
	PP*	-	3.3 ± 0.5 (86)	3.4 ± 0.4 (69)	0.1 ± 0.3 (69)	3.3 ± 0.5 (268)	3.3 ± 0.5 (166)	0.04 ± 0.3 (165)	**0**.**09** (**0**.**003** - **0**.**2**), ***p****=****0****.****049***
**Diet quality – Fibre score (± SD)**	ITT	p = 0.64	2.8 ± 0.7 (174)	2.9 ± 0.6 (109)	0.1 ± 0.3 (108)	2.7 ± 0.7 (180)	2.8± 0.7 (126)	0.1 ± 0.4 (126)	0.06 (−0.04-0.2), *p = 0.24*
	PP	-	2.9 ± 0.6 (86)	3.0 ± 0.6 (69)	0.1 ± 0.4 (69)	2.7 ± 0.7 (268)	2.8 ± 0.7 (166)	0.06 ± 0.4 (165)	0.07 (−0.05 - 0.2), *p = 0.25*
**Diet quality – Total score (± SD)**	ITT	p = 0.89	3.1 ± 0.5 (174)	3.2 ± 0.5 (109)	0.1 ± 0.3 (108)	3.1 ± 0.5 (180)	3.2 ± 0.4 (126)	0.1 ± 0.3	0.04 (−0.03 - 0.1), *p = 0.31*
	PP*	-	2.1 ± 0.5 (86)	2.5 ± 0.4 (69)	0.1 ± 0.3 (69)	1.5 ± 0.4 (268)	2.1 ± 0.4 (166)	0.05 ± 0.3 (165)	**0**.**09** (**0**.**009** - **0**.**2**), ***p****=****0****.****027***
**Weekly minutes of physical activity** (**Median**; **IQR**)	ITT	*p = 0.33*	150 (45,270) (177)	190 (100 , 330) (110)	11.2 ± 196.3 (109)	150 (60, 300) (182)	130 (60, 279) (127)	−15.8 ± 214.8 (127)	+**27**.**0** (−**26**.**1** - **80**.**1**), ***p****=****0****.****32***
	PP	-	180 (90, 300) (87)	230 (120, 330) (165)	+11.5 ± 195.5 (71)	150 (40, 300) (272)	130 (60, 277) (72)	−9.7 ± 211.3 (165)	+**21**.**3** (−**36**.**5** -**79**.**1**), ***p****=****0****.****47***
**Intention to breastfeed** (**Median**; **IQR**)	ITT	*p= 0.30*	14 (10,16) (172)	15 (12.5, 16) (118)	0.8 ± 3.1 (99)	13.5 (10, 15.5) (168)	15 (12,16) (105)	0.9 ± 2.3 (114)	−0.06 (−0.8 - 0.7), *p = 0.88*
	PP	-	14.8 (12,16) (82)	15.5 (13,16) (69)	0.7 ± 2.8 (65)	13.5 (10, 15.5) (258)	14.7 (11.5, 16) (154)	0.9 ± 2.7 (148)	−0.3 ( −1.1 - 0.5), *p = 0.43*

* significant difference between groups over time; Differences were examined with independent group t-tests (for normally distributed variables) and Mann U Whitney tests (for baseline non-parametric variables).

### Dietary intake

#### Fruit and vegetables

Significant between-group differences were observed in the percentage change over time of women meeting the fruit guidelines (ITT) and fruit and vegetable guidelines (PP) for pregnancy (Table 2). At baseline, women consumed approximately half of the recommended servings of fruit per day (HSP: 1.9 ±1.2, UC: 1.8±1.2) and vegetables per day (HSP: 2.5±1.3, UC: 2.3±1.3). The between-group difference in change over time in servings of fruit and vegetables consumed between the two sites was approximately a quarter of a serve per day, but not significant (ITT). However, both were significant in the PP analysis (Table 3). Fruit and vegetable intake increased in women who attended the HSP workshop by almost a half a serve per day.

#### Diet quality index

Changes in fat, fibre and overall diet quality index scores were not significant in the ITT analysis. Significant between-group differences were observed in the fat and overall diet quality index scores in the PP analysis (Table 3). The diet quality index improved over time in the women who attended the HSP workshop.

#### GWG awareness

Significantly more women who attended the HSP intervention could report correct (exact or rounded) GWG goals compared with those who only received the booklet (8% difference). This was not significant in the ITT analysis (Table 2).

#### Physical activity

Weekly minutes of PA was positively skewed and was therefore reported as median and interquartile range (IQR). Between-group difference in change in weekly median minutes of PA over time was clinically relevant, but not statistically significant for both ITT and PP analyses (+27 minutes/week and +21.3 minutes/week, respectively) (Table 3). There was not sufficient statistical power to detect a significant between-group difference of this magnitude (319 women required per group to detect 30 minute difference over time [28,29]).

#### Cigarette smoking

Approximately 1-2% of women in each group quit smoking when they became pregnant. By 12-weeks post-service-entry, a further percentage had quit smoking. However there was not a significant difference between groups (Table 2); further, there was not sufficient power to detect this small change.

#### Intention to breastfeed

Intention to breastfeed was negatively skewed and was therefore reported as median and the corresponding IQRs. No significant between-group difference was observed in women’s intention to breastfeed (ITT or PP) (Table 3); these score were high at both time points for both groups, with a score range being 0 (low) to 16 (high) [38].

#### Comparison of HSP attenders with ‘Booklet’ group (non-attenders and usual care women)

Women who attended the HSP (PP analysis) differed on some demographic and behavioral measures. The attendees were older (30.7 ± 4.8 vs 28.8 ± 4.9 years, *p = 0.002*), consumed more serves of vegetables daily (2.7 ± 1.4 vs 2.3 ± 1.2 serves, *p = 0.009*), had a higher total fibre score on the diet quality index (2.9 ± 0.6 vs 2.7 ± 0.7, *p = 0.012*) and had a higher median BF intention (14.8 (IQR 12–16) vs 13.5 (10–15.5). Similar proportions of non-smokers were in both groups, however fewer smokers attended HSP (non-smoker 27% vs 23%; smoker 11.7% vs 36.7%, *p = 0.014*). Education status of attendees was higher than non-attendees/booklet group (less than year 12 – 12.4% vs 34.3%, trade/apprenticeship/diploma – 21.5% vs 27.1%, degree or higher – 35.4% vs 17.7%), *p = 0.001*.

## Discussion

This study’s aim was to evaluate the effectiveness of a low intensity, early antenatal health promotion workshop (*The Healthy Start to Pregnancy; HSP*) in improving lifestyle behaviors associated with demonstrated maternal and infant health outcomes. Unfortunately, assessment of many aspects of the ITT analysis is limited by lower than ideal attendance at the scheduled HSP sessions in women randomised to this approach. In the ITT analysis, only the proportion of women meeting fruit guidelines achieved statistical significance. The change in minutes of PA undertaken approached hypothesised difference, but the study was not powered for this outcome. The ‘quarter serve’ increases observed in daily fruit and vegetable intakes are also clinically relevant. Furthermore, “per protocol” attendance at the *HSP* workshop resulted in improvements in important health behaviors. Outcomes from exposure to the intervention met most of the primary hypotheses and changes were clinically relevant. Secondary outcomes not met were smoking cessation levels, which did decrease, but did not demonstrate a between group difference. This population already had a high intention to breastfeed.

The strong theoretical basis underpinning this program (the 5As and activities to improve self-efficacy) may explain the behavior change observed. This work extends the concept of the ‘*Pregnancy Pocketbook*’, a woman-held pregnancy resource, delivered by midwives to improve health behaviors, designed and delivered according to the 5As [27]. Delivery of this low intensity intervention resulted in a similar clinically relevant increase in PA between groups over time (+20 minutes). All women increased fruit and vegetable intake, possibly due to a concurrent statewide campaign. More recently, Jackson et al’s (2011) low-intensity early antenatal RCT intervention (using a different delivery method, but similar sample size, content and theoretical basis) demonstrated a similar increase in PA (+28 minutes), as well as increased fruit and vegetable intake (+0.44 serves, combined) and an improvement in diet quality [46] and these changes also reflect earlier behavior change strategies in non-pregnant populations to increase fruit and vegetable intake [47,48]. Modest, but clinically relevant behavioral changes are possible with appropriately designed interventions.

Promisingly, significantly more women who attended the HSP were aware of the GWG goals. Clear evidence exists that women are more likely to experience correct GWG if provided (correct) advice [6,7] and clinical guidelines recommend this is a key element for women in the obese BMI category [49].This requires consistent and clear messages across all health professionals.

Strengths of this intervention include the theoretical framework underpinning the intervention [50], the RCT methodology and good participant retention. There is clear evidence that self-monitoring and goal setting helps regulate successful behavior change and are essential in any healthy lifestyle program [22,51]. Further, our population appeared to be representative of other Australian antenatal populations [15,16], including that of the study hospital [14], strengthening the generalizability of the results. However, the differing characteristics of the groups in the PP analysis may limit the generalizability of these findings.

Whilst the low attendance at the HSP intervention attendance is an important limitation of our study, women who were exposed to the program experienced significant behavioral changes. This highlights the need to investigate barriers to service engagement, as well as addressing service access issues. Women who declined study involvement and/or fed back reasons for non-attendance mentioned practical problems with accessing a large, inner city hospital (especially parking) and getting time off work to attend a workshop (which, being run within existing department resources, was only available one morning, one afternoon and one evening a week). Further work improving engagement with hard-to-reach women, perhaps through offering a suite of (effective) delivery methods (E.g. e-health) may overcome program access problems. This could be informed by a follow-up qualitative study of our study non-attenders. Any program must include ongoing contact, demonstrated to be required for any effective behavior change in pregnant and non-pregnant populations [52].

Another limitation may be the use of self-report measures, introducing the potential for under-reporting of behaviors or weight. It has been suggested women from higher BMI ranges under-report their weight which may have resulted in under-reporting in this study [53]. However, it has been suggested this is minimal [54] and the proportions identified in our study reflected the wider hospital population [14]. Further, the fruit and vegetable assessment tool provides reasonable rank-order validity, but quantities reported tend to differ in absolute terms (over estimation) [35,36]. However, we report change over time between groups, so whilst outcomes may be conservative, the relative changes between groups remain.

Despite the positive changes observed in this study, the low prevalence of women meeting fruit, vegetable and PA guidelines and knowledge of GWG goals is a concern. Sufficient fruit and vegetable intake may be the most important public health message for the decrease of chronic disease [55]. Jackson et al. ( [1999]) emphasises the importance of improving diet quality and health behaviors, rather than modifying diet for the sole purpose of 'managing GWG', as there are clear public health benefits of adherence to diets that align with national dietary guidelines, beyond the causal links to GWG [43]. Further importance of diet quality is realised with Rifas-Shiman et al's. ( [2009]) demonstration that early pregnancy diet quality is associated with lower blood glucose levels at GDM screening and lower risk of pre-eclampsia [56]. Simple, positive messages, such as 'increasing fruit and water intake' may result in decreased (displaced) take away and soft drink intake [12], with resultant health benefits.

Further, as PA may remain low after pregnancy, and insufficient postnatal PA has been associated with weight retention [57], poor mental health [13], as well as increasing chronic disease risk [5], ongoing intervention is required to improve women's health behaviors at this time. Barriers to PA must be recognised and addressed when delivering programs. Evenson et al. (2009) provides ideas and opportunities, noting that 85% of barriers are interpersonal, especially health- related, including women's concern with pregnancy complications, as well as non-health ones, such as low motivation, time, childcare, lack of knowledge and enjoyment [58].

Antenatal health promotion programs should be delivered by appropriately skilled health professionals and continue to incorporate emerging evidence about content, format, behavioral predictors, and group-accessibility to ensure maintenance of an effective and efficient woman-focused service. Raising awareness and priority of the importance of health behavior improvement (to health professionals and women) is required to ensure consistent messages are delivered and reinforced by all maternity care providers.

## Conclusions

This research evaluated the effectiveness of a low-intensity early antenatal health promotion workshop on lifestyle behaviors and knowledge with demonstrated maternal and infant health outcomes. Per protocol attendance at the HSP resulted in significant improvements in a suite of health behaviors. Further investigation and potential service changes are required to facilitate women's workshop attendance, as well as investigate alternate methods of supporting women, as many still do not meet pregnancy-lifestyle recommendations. More broadly, antenatal lifestyle interventions should include a theoretical basis to facilitate beahvior change and should promote improvement of diet quality, rather than just ‘managing GWG’, and these healthy lifestyle messages need to be consistently reinforced by all maternity care providers.

## Abbreviations

ANC: Antenatal Clinic; ATSI: Aboriginal and/or Torres Strait Islander; BMI: Body Mass Index; EB: Evidence based; GDM: Gestational Diabetes Mellitus; GWG: Gestational Weight Gain; HSP: Healthy Start to Pregnancy; IOM: Institute of Medicine; IQR: Interquartile range; ITT: Intention to Treat; MH: Maternity Hospital; PA: Physical Activity; PP: Per Protocol; UC: Usual Care.

## Competing interests

The author(s) declare that they have no competing interests.

## Authors’ contributions

SW conceived of and developed the study (design and all content materials), analysed and interpreted the data and drafted the manuscript. HDMc analysed and interpreted the data and helped draft the manuscript. Both authors read and approved the final manuscript.

## Authors’ information

SW is an Advanced Accredited Practising Dietitian and has Honours and a PhD in psychology. She is the senior maternal health dietitian at the Mater Mothers' Hospital, Brisbane. She is a program leader in the Mater Medical Research Institute's (MMRI) Mothers and Babies theme, 'Optimising outcomes for mothers and babies at risk'. She is supported by an 'NHMRC TRIP Fellowship' (National Health and Medical Research Council Translating Research Into Practice). HDMc is the Head of the University of Queensland Medical School, Director of Obstetric Medicine (Mater Mothers’ Hospital) and MMRI Theme Leader (Mothers and Babies).

## Support

Trial funded from JP Kelly Foundation, Mater Health Services.

## Pre-publication history

The pre-publication history for this paper can be accessed here:

http://www.biomedcentral.com/1471-2393/12/131/prepub
